# Measuring reader fatigue in the interpretation of screening digital breast tomosynthesis (DBT)

**DOI:** 10.1259/bjr.20220629

**Published:** 2023-01-12

**Authors:** Yan Chen, Ellhia S Sudin, George JW Partridge, Adnan G Taib, Iain T Darker, Peter Phillips, Jonathan J James, Keshthra Satchithananda, Nisha Sharma, Michael J Michell

**Affiliations:** 1 University of Nottingham, School of Medicine, Translational Medical Sciences, Clinical Sciences Building, City Hospital Campus, Hucknall Road, Nottingham, United Kingdom; 2 Health and Medical Sciences Group, University of Cumbria, Lancaster, United Kingdom; 3 Nottingham University Hospitals NHS Trust, Nottingham Breast Institute, City Hospital Campus, Hucknall Road, Nottingham, United Kingdom; 4 Department of Breast Radiology, National Breast Screening Training Centre, King’s College Hospital, Denmark Hill, London, United Kingdom; 5 Leeds Breast Screening Unit, Leeds Teaching Hospital, York Road, Leeds, United Kingdom

## Abstract

**Objectives:**

The interpretation of digital breast tomosynthesis (DBT) screening examinations is a complex task for an already overstretched workforce which has the potential to increase pressure on readers leading to fatigue and patient safety issues. Studies in non-medical and medical settings have suggested that changes in blink characteristics can reflect fatigue. The purpose of this study is to investigate the use of blink characteristics as an objective marker of fatigue in readers interpreting DBT breast screening examinations.

**Methods:**

Twenty-six DBT readers involved in the UK PROSPECTS trial interpreted a test set of 40 DBT cases while being observed by an eye tracking device from November 2019 to February 2021. Raw data from the eye tracker were collected and automated processing software was used to produce eye blinking characteristics data which were analysed using multiple linear regression statistical models.

**Results:**

Of the 26 DBT readers recruited, eye tracking data from 23 participants were analysed due to missing data rendering 3 participants’ data uninterpretable. The mean reading time per DBT case was 2.81 min. There was a statistically significant increase in blinking duration of 0.38 ms/case as the reading session progressed (*p* < 0.0001). This was the result of a significant decrease in the number of ultra-short blinks lasting ≤50 ms (*p* = 0.0005) and a significant increase in longer blinks lasting 51–100 ms (*p* = 0.008).

**Conclusion:**

Changes in blinking characteristics could serve as objective measures of reader fatigue and may prove useful in the development of DBT reading protocols.

**Advances in knowledge:**

Blink characteristics can be used as an objective measure of fatigue; however there is limited evidence of their use in radiological settings. Our study suggests that changes in blink duration and frequency could be used to monitor fatigue in DBT reading sessions.

## Introduction

Breast cancer screening has been shown to reduce breast cancer mortality.^1,2^ In England, over 2 million women were screened in the 12 month period ending March 2020, representing an 18.3% increase over the preceding 10 years.^3^ Furthermore, the UK radiology workforce census highlights an increasing shortage of radiologists, with breast imaging being particularly vulnerable.^4^

In many countries, digital breast tomosynthesis (DBT) is being introduced into mammographic screening practice as it has been shown to improve cancer detection rates and reduce false-positive interpretations, leading to a reduction in recall rates.^5^ DBT produces a volumetric reconstruction of the whole breast, but viewing and interpreting the multiple image slices of each breast in each projection requires around a doubling of interpretation time compared to FFDM.^6–8^ In the UK, a trial (PROSPECTS) is currently underway to evaluate the implementation of DBT within the National Health Service Breast Screening Programme (NHSBSP).^9^

The increase in the number of breast screening examinations, the complexity of the screening interpretation task and the looming workforce crisis has the potential to place additional pressure and stress on readers endeavouring to keep pace with the demands of the service. As well as the detrimental effects this has on wellbeing and morale, fatigue in overworked readers can adversely impact on patient safety through possible diagnostic errors. Early eye tracking studies have identified visual search behaviours as a potential cause of diagnostic errors.^10,11^ Fatigue has been shown to affect visual search patterns, which could hence affect diagnostic interpretation.^12^ Diagnostic errors contribute to a significant number of preventable deaths in English NHS hospitals.^13^

Modern eye tracking technology allows the non-intrusive contemporary monitoring of eye movements and related visual search parameters. Involuntary blinking is not solely dependent on eye dryness; evidence suggests it is linked to cognitive load and fatigue. Indeed a greater blink duration is associated with increased levels of mental workload and fatigue.^14–16^ In contrast, the evidence for blink frequency and fatigue is mixed.^15,17,18^

The evidence exploring how blinking can be an objective measure of fatigue in a radiological setting is scarce. With the potential significant, but preventable harm related to fatigue during image interpretation and its correlation to blink characteristics a greater emphasis must be placed on research to facilitate its clinical application. Particularly with the ease in which modern technology can record blink data. Therefore, the present study aims to assess the use of blink characteristics as a marker for the onset of fatigue in readers interpreting DBT screening examinations in the NHSBSP as part of the PROSPECTS study.

## Methods

### Participants

Breast screeners participating in the PROSPECTS trial from three different NHSBSP screening centres were invited to take part. The PROSPECTS study is a prospective randomised trial of DBT plus standard 2D FFDM, compared to standard FFDM alone in breast cancer screening; ClinicalTrials.gov Identifier: NCT03733106.^9^ The trial has London - Dulwich Research Ethics Committee approval and as a result approval granted for the eye-tracking portion of the study. 26 trained readers (consisting of radiologists and radiographers) provided written consent and participated in this study from November 2019 to February 2021. Radiographers make up around half the readers in the UK screening programme and are trained to Master’s level or equivalent. The NHSBSP requires all readers to interpret at least 5000 mammograms per year, with double reading the standard of care.^19^ The study was undertaken using approved COVID-19 precautions.

The eye tracking data from 17 of the 26 participants in this study were previously analysed,^20^ however, the analysis was limited and has been expanded here, providing a ‘per case’ breakdown. The 26 participants analysed in this study form a subset of a previously published and more recent study.^21^ In Partridge et al. 2022, a greater sample of readers were incorporated owing to further data collection since the analysis of this study. Furthermore, a different blink detection algorithm was applied to the raw data in this iteration to account for the noise observed in some of the earlier datasets. The analysis here assesses changes in eye blink behaviour across the entire reporting session in a per case fashion and relates this to diagnostic performance. This was not undertaken in the previously published Partridge et al. 2022 study.

### DBT images & image display apparatus

40 de-identified DBT cases, acquired as part of the PROSPECTS trial, were used to make up a reading set for the study. The test-set was heavily enriched with biopsy proven cancers (47.5% malignant, 12.5% benign and 40% normal). The cases were selected by a breast radiologist with more than 20 years’ experience in a non-consecutive manner to encompass a wide variety of difficulties and lesion types for abnormal cases.

**Figure 1.**
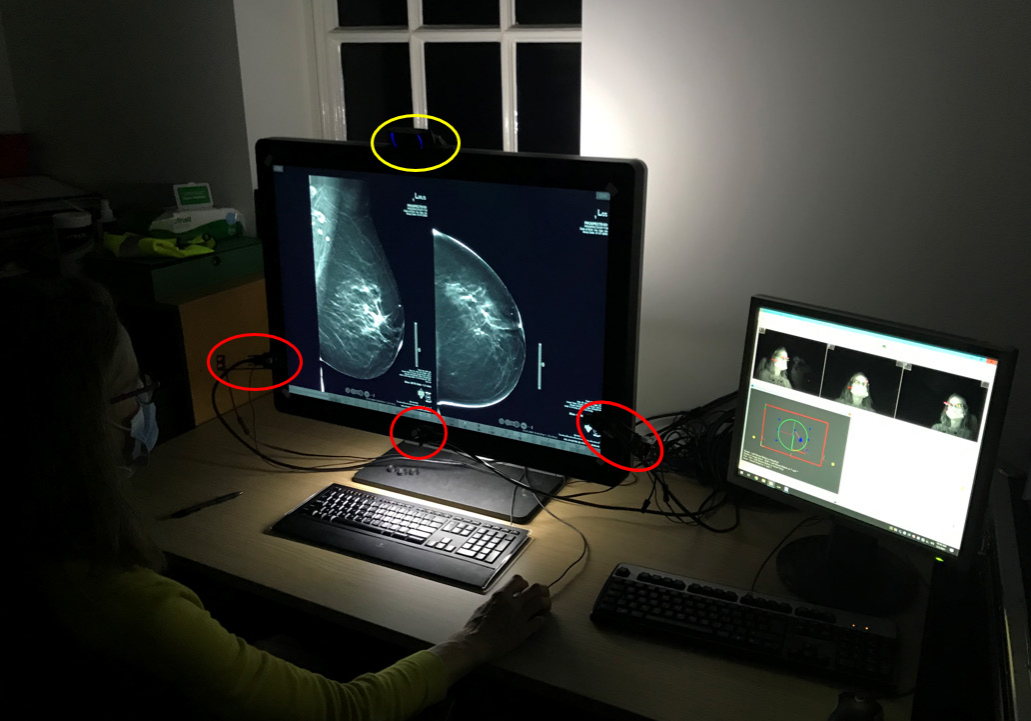
An example of the experimental set-up showing the non-intrusive eye tracking cameras (inferior 3 circles) and the scene camera (single superior circle) positioned on a participant workstation. The monitor to the right was used for eye tracking calibration and monitoring during the experiment.

Participants individually viewed the images on a Hologic Securview workstation (Hologic, Inc.) with high resolution mammography approved monitors. Cases were presented to each participant in a different, random order, to reduce the confounding effect of case mix and difficulty across the reading set. A separate computer controlled the eye tracking system. The equipment was set up in the participants’ usual workstation area, to emulate natural reading habits and visual search behaviour (Figure 1). The participants were aware of the total number of cases to read and the number of cases remaining as the reading session progressed. Readers were blinded to any pathology or outcome data for each case. Readers knew the study involved eye-tracking, but were unaware as to which metrics were being measured.

### Eye tracking device

A non-intrusive eye tracking device, SmartEyePro (Smart Eye AB, Gothenburg, Sweden), was used in this study. This eye tracker, comprised of three small cameras, monitored participants’ visual search behaviour at a sampling rate of 60 Hz. An additional scene camera was placed on top of the participant workstation to record the reader’s behaviour and help identify any instance where data were lost (Figure 1). Prior to each data collection, the eye tracking cameras were adjusted to suit each participant’s head position and calibrated to ensure accurate identification of each participant’s facial features to guide the eye tracking system. Coupled with the eyesDX software application suite (eyesDX), participants’ eye tracking data, workstation screen capture and the scene camera recording were automatically recorded and compiled in real-time.

### Procedure

A pre-set hanging protocol was used to maintain standardisation. Participants examined each DBT case and verbally reported their decisions about both case images as either normal, benign, indeterminate, suspicious or highly suspicious, as well as indicating the three-dimensional locations of any abnormalities on the images. All decisions were recorded by a research assistant using the PERFORMS online reporting software. Verbal reporting was opted to minimise interruption to the eye tracking recording, avoiding participants having to look away from the workstation to record findings themselves. This method has been utilised effectively in a previous experiment of a similar nature.^21^ Recordings were taken as part of a normal working day between 8am and 5pm except for one candidate whom started their session at 7:30am. Those which undertook the majority of their session before 12pm and after 12pm were classified as ‘morning’ and ‘afternoon’ participants respectively.

The experimental procedure was paused after approximately every 40 min to offload the large eye tracking data files to an external hard drive due to local storage capacity limits. This process took a median time of 6.1 min (IQR 2.8–10.8) during this time participants were asked to remain in the room.

### Blink detection software

Due to the large and long duration datasets, blink detection software was developed and manually validated as described.^22^ The software allowed automatic identification of eye blink events and their durations using the raw participant eyelid opening data recorded by the eye trackers; where a ‘blink’ event was considered as a ‘near closure’ of the eyelid (*i.e.,* eyelid opening ≤4 mm), based on previous calculations and validations.^22^ This automated data processing ensured that human bias and error were eliminated from this stage.

Similar to other studies, we found that blinks have variable duration, hence they can be divided into several subcategories.^23^ We analysed the raw blink data and classified the participants’ blinks into four classes based on findings in this study and existing literature^24^: ultra-short blinks (≤50 ms); short blinks (51–100 ms); long blinks (101–500 ms), and microsleeps (>500 ms).

### Data analysis

Blink data were analysed using multiple linear regression statistical models, with *Participant ID* and *Case ID* set as random effects. The α-level for statistical significance was set at 0.05 for all analyses. Statistical calculations were conducted using R v. 4.0.5 (R Foundation for Statistical Computing). Participants with large volumes of missing data were omitted from the analysis.

## Results

### Participants’ characteristics

The 26 participants consisted of 19 board-certified consultant radiologists, 4 consultant radiographers and 3 advanced practitioners. Data analysis was performed on 23 DBT readers. Three participants were excluded from the study due to technical issues during the experiment resulting in unreliable data. Two participants had incomplete data, but were still included in the analysis (30 and 45% of data were lost for participants 9 and 19, respectively; calculated as a percentage of read cases with missing data). Reader characteristics are shown in Table 1. Of the 21 participants with complete data, there was 39.3 h of recordings. Reading time was tested for normality using the Kolmogrov–Smirnov statistic; *p* = 0.9 indicating a normal distribution. The mean completion time of all 40 DBT cases was 112.3 min (95% CI: 93.4–131.3 min, *n* = 21). The mean reading time per case took on average 2.81 min (95% CI: 2.33–3.28 min, *n* = 21) across these participants.

**Table 1.** Participant DBT experience and DBT reading time

Participant	Number of years of DBT experience	Total reading time for 40 cases (min)	Average reading time per case (min)
1	3	102.3	2.6
2	3	135.1	3.4
3	3	173.8	4.3
4	5	68.7	1.7
5	5	143.4	3.6
6	4	114.5	2.9
7	3	172	4.3
8	2	182.4	4.6
9	2	N/Aa	N/Aa
10	0	162.1	4.1
11	0.5	114.5	2.9
12	1	59.8	1.5
13	1	110.7	2.8
14	1	105.3	2.6
15	6	115.1	2.9
16	4	60.5	1.5
17	6	35.6	0.9
18	6	60.8	1.5
19	1.5	N/Aa	N/Aa
20	2	121.1	3
21	0	104	2.6
22	3	125.6	3.1
23	1.5	91.9	2.3

DBT, digital breast tomosynthesis.

a N/A is listed for the two participants with incomplete data. 30 and 45% of data were lost for participants 9 and 19, respectively (calculated as a percentage of read cases with missing data).

10 participants completed their session in the morning, with a mean trial duration of 154.1 min (95% CI: 117.3–190.8 min). The afternoon mean trial duration was lower, at 120.2 min (95% CI: 95.0–145.5 min), however, this was not significant (*p* = 0.140).

DBT experience among participants is shown in Table 1. DBT reading experience ranged from 0 to 6 years (median = 3 years; IQR = 2.75 years).

### Blink rate and duration

#### Overall

**Figure 2.**
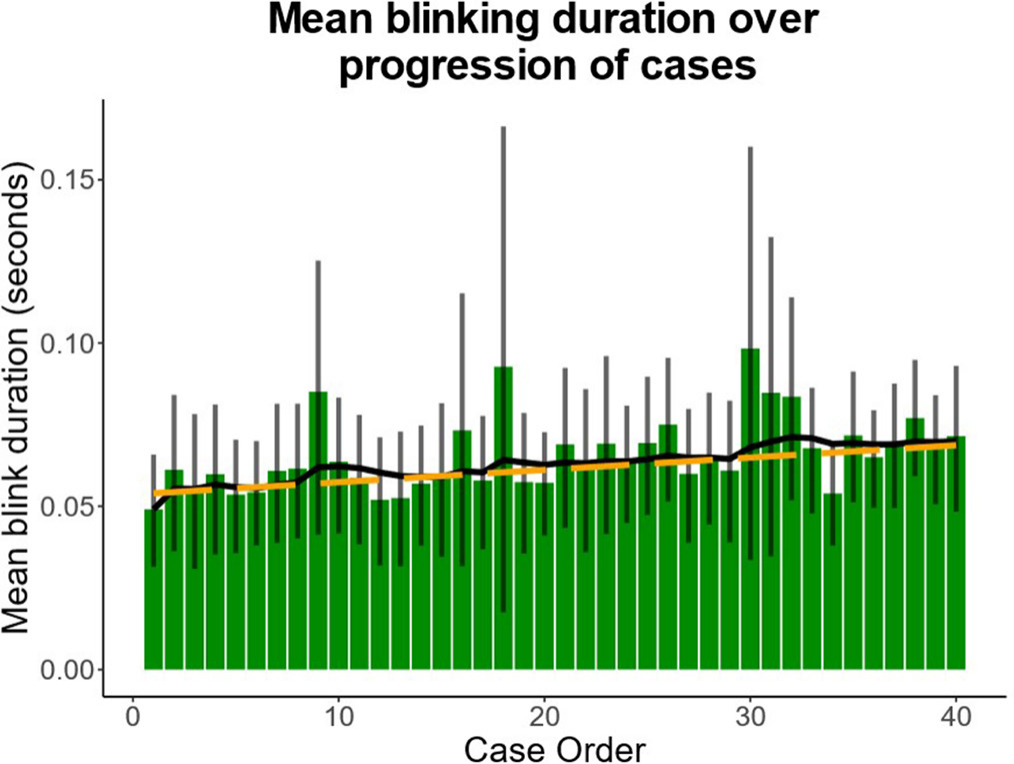
Change in average blink duration over case progression. Blink duration significantly increased by 0.38 ms/case (*p* < 0.0001) as the reading session progressed. Bars represent the mean blink duration per case. Error bars denote 95% confidence interval. Solid line shows exponential moving average. Dotted line shows regression line.

The average blink duration recorded during the first case of the reading session was 49.05 ms (95% CI: 31.50–65.86 ms). A linear mixed-effects regression showed a statistically significant increase in blinking duration by 0.38 ms/case (95% CI: 0.20–0.55 ms/case; *p* < 0.0001) as the reading session progressed (Figure 2).

**Figure 3.**
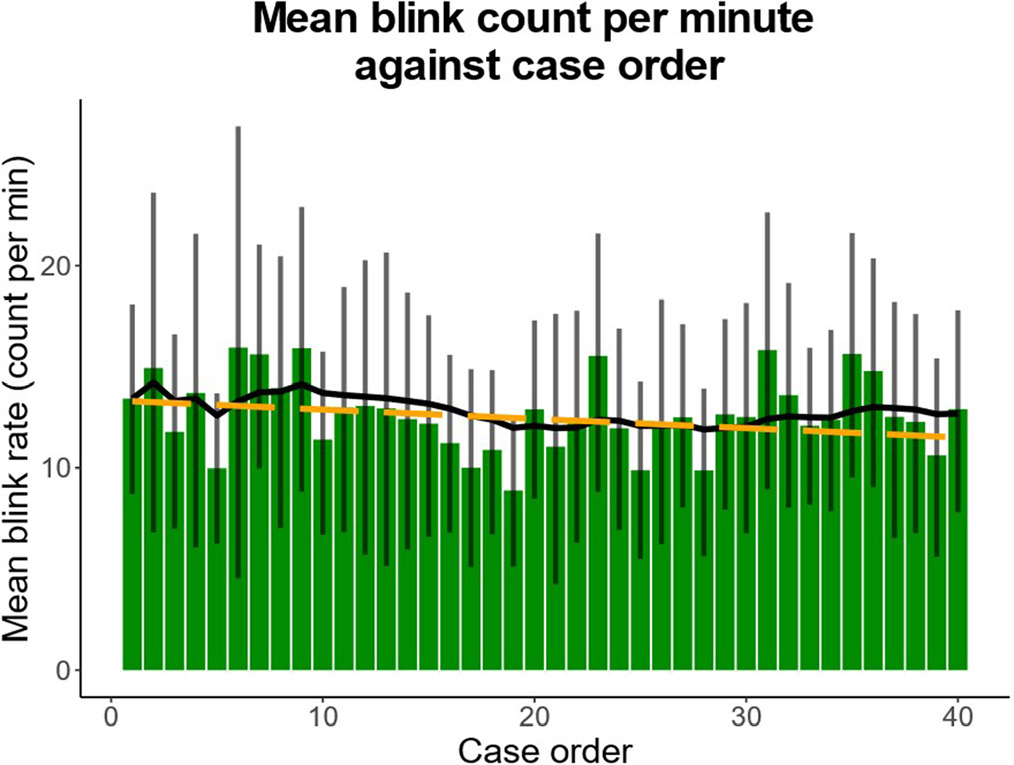
Average blink count per minute over case progression. Blink frequency decreased non-significantly as reading session progressed (−0.05 blinks/min/case, *p* = 0.089). Bars indicate mean blink rate per case. Error bars denote 95% confidence interval. Solid line represents exponential moving average. Dotted line predicts rate by linear regression.

The average blink rate during the first case of the reading session was 13.44 blinks/min (95% CI: 8.71–18.10 blinks/min). There was a non-significant trend towards decreasing blink rate as the reading session progressed (change in blink rate: 0.05 blinks/min/case, 95% CI: −0.10 to 0.01 blinks/min/case; *p* = 0.089; Figure 3).

#### Blink characteristics

**Figure 4.**
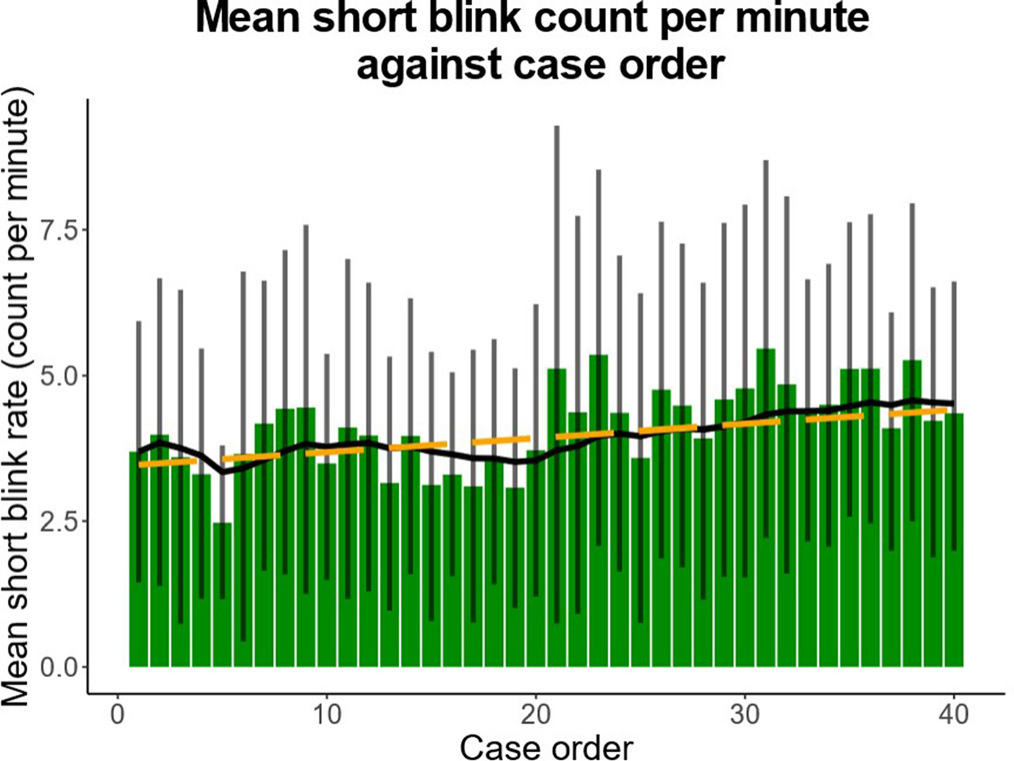
Mean short blink (51–100 ms) count per minute over progression of cases. There was a significant increase in short blinks with session progression (0.024 blinks/min/case, *p* = 0.008). Bars indicate mean short blink rate per case. Error bars denote 95% confidence interval. Solid line represents exponential moving average. Dotted line predicts rate by linear regression.

**Figure 5.**
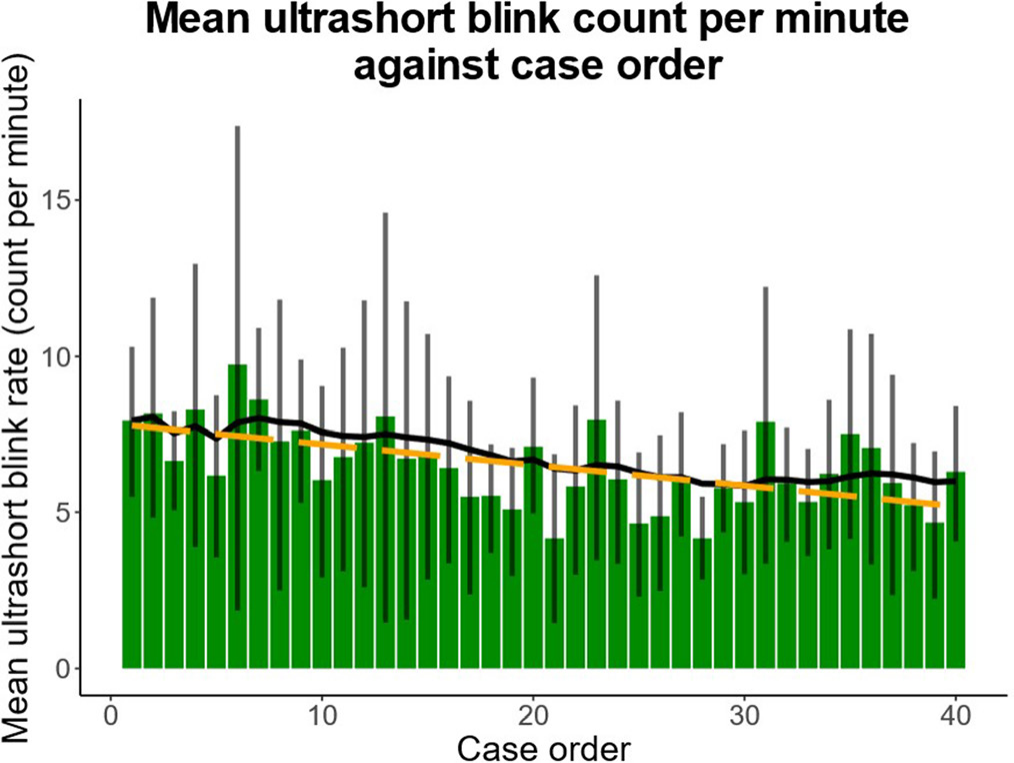
Mean ultrashort blink (≤50 ms) count per minute over progression of cases. Ultrashort blink frequency decreased as reporting session progressed (−0.066 blinks/min/case, *p* = 0.0005). Bars indicate mean ultrashort blink rate per case. Error bars denote 95% confidence interval. Solid line represents exponential moving average. Dotted line predicts rate by linear regression.

Linear mixed-effects regression showed a significant increase in short blinks (blink duration of between 51 and 100 ms) with case progression (0.024 blinks/min/case, 95% CI: 0.006 to 0.042; *p* = 0.008; Figure 4). There was also a significant decrease in ultrashort blinks (blink duration ≤50 ms) with case progression (−0.066 blinks/min/case, 95% CI: −0.104 to −0.029; *p* = 0.0005; Figure 5).

Blinking subcategories and their changes with case progression were analysed. There were no significant changes in the rate of longer blinks of between 101 and 500 ms, or of the longest blinks of >500 ms. Table 2 summarises the effect seen on blink characteristics during the reading session.

**Table 2.** Summary of changes in blink rate for each subcategory of blink over reading session using mixed effect linear regression

Blink categories	Duration	Effect size	95% CI	*p*-value
Microsleep (log-rate)	>500 ms	−0.094	−0.022 to 0.004	0.158
Long blink	101–500 ms	−0.046	−0.099 to 0.007	0.090
Short blink	51–100 ms	0.024	0.006 to 0.042	**0.008^a^**
Ultrashort	≤50 ms	−0.066	−0.104 to −0.029	**0.0005^a^**

a Bolded *p*-values highlight significance.

### Diagnostic accuracy against case order

**Figure 6.**
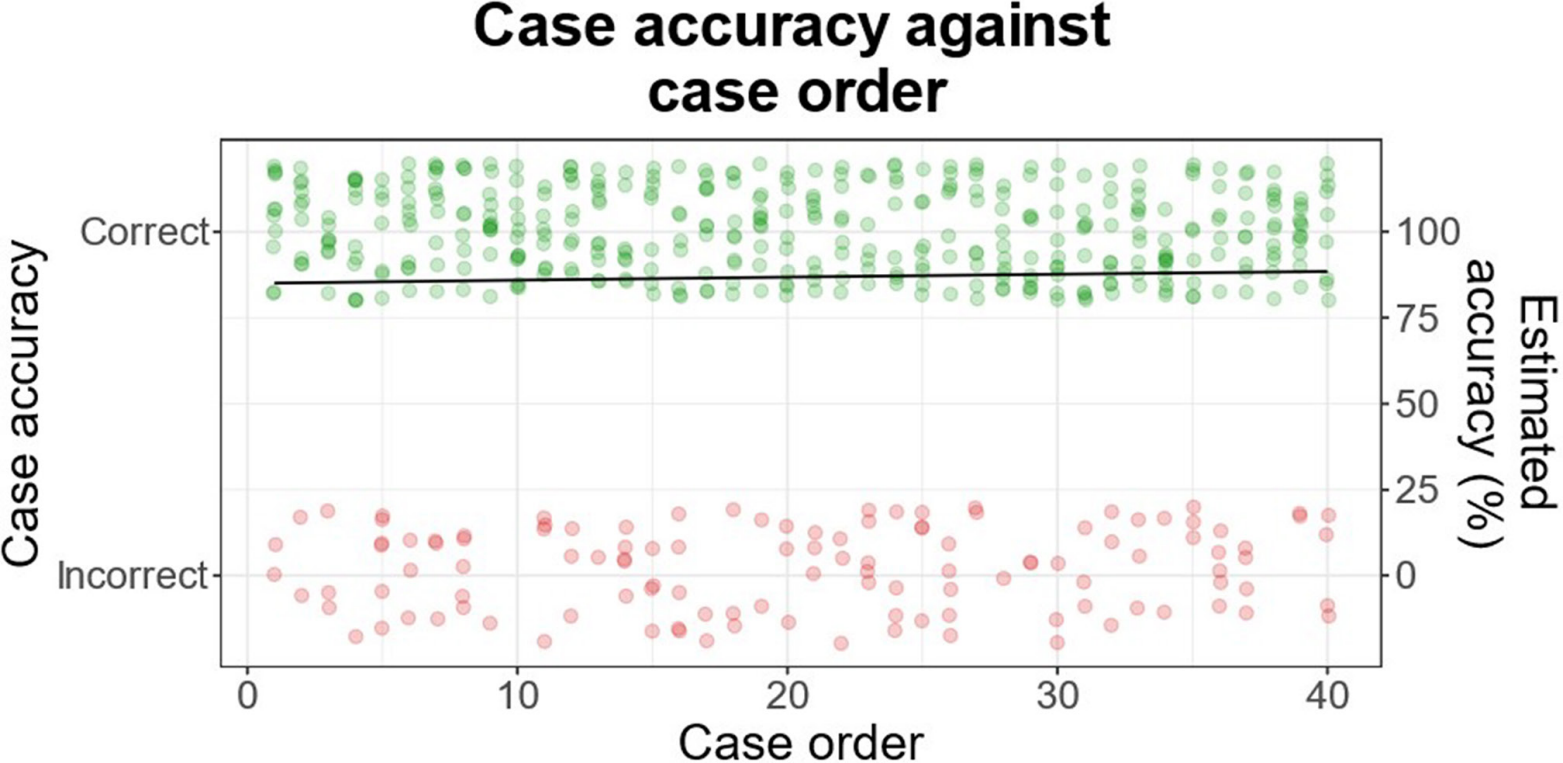
Tabulation of cases based on diagnostic accuracy against case order. There was no significant change in diagnostic accuracy as reporting progressed (*p* = 0.491). Superior dots represent correct cases, whereas inferior dots represent incorrect cases. The line shows the estimated accuracy by logistic regression.

Any association between session progression and performance (as measured by diagnostic accuracy) was investigated. A logistic mixed-effects regression found no significant effect of case order on diagnostic accuracy (log-odds: 0.008, 95% CI: −0.014 to 0.029; *p* = 0.491; Figure 6).

## Discussion

Eye blinking has been shown to reflect fatigue, but there has been little work in medical imaging. In this study of 23 readers, reporting a set of 40 DBT screening cases, the average blink duration significantly increased with progression in reading sessions (0.38 ms per case, *p* < 0.0001), and there was a trend towards decreasing blink rate (0.05 blinks per minute per case, *p* = 0.089). The increase in blink duration was explained by a significant decrease in the number of ultra-short blinks (≤50 ms, *p* = 0.0005) and a significant increase in short blinks (51–100 ms, *p* = 0.008). Total time to complete a reading session can be used as a surrogate for fatigue as explored in a previous study analysing radiological diagnostic accuracy dependent on shift time.^25^ Interestingly, in our study, there was no significant difference in the trial times between morning and afternoon sessions.

The eye is particularly prone to the effects of fatigue. Prior studies have measured changes in the ability of the eye to focus and accommodate, but there is limited evidence investigating changes in blink characteristics in a radiological setting. A single study measuring eye fatigue via blink characteristics as a dependent variable of room illumination is noted.^26^ There have been studies in the transport industry, where tiredness can precipitate a fatal road traffic collision, highlighting an association between blink nature and fatigue. Solerimanloo *et al* (2019) observed fatigue in drivers manifesting as an increase in blink duration, which ran parallel to impairment in driving performance.^14^ Furthermore, a review article by Cori *et al* (2019) highlighted how blink duration increased with fatigue across various drowsiness levels, experiments and acquisition methodologies suggesting it was a robust measure of tiredness.^15^Therefore, the significant increase in blink duration observed in the screening DBT readers in our study is likely to be a manifestation of visual fatigue.

This study found an overall non-significant trend towards decreased blink rate with case progression. This is consistent with findings by Divjak *et al* (2009) and Zheng *et al* (2012),^17,27^ although there is a lack of consensus within the existing literature regarding blink rates and fatigue.^15^ *Divjak et al* (2009) argued that an increase in blink rates with fatigue is due to participants trying to fight fatigue by doing short bursts of rapid blinking,^27^ which could explain the significant increase in the rate of short blinks in our study. Here, we demonstrated that blinks (lasting 51–100 ms) increased significantly with case progression, whereas the rate of ultrashort blinks (≤50 ms) decreased significantly with case progression.

There is evidence that radiology reporting tasks are negatively affected by fatigue and this can translate into an increase in error rates. Several studies have demonstrated an increase in discrepancy rates in radiology reporting between radiology residents and consultant radiologists over the course of the working day and week and during on call shifts.^28,29^ Furthermore, case load and reporting speeds can significantly impact diagnostic accuracy. Population breast cancer screening is a high-volume activity and the current workforce crisis in the UK leads to inevitable pressure on reporting speeds. Screening DBT is a higher complexity examination to interpret as compared to conventional FFDM, resulting in significant increases in reading time.^7,8^ DBT reporting times in our study are in line with those reported in the literature, with an average interpretation time of 2.81 min. Dang et al also recorded an average reporting time of 2.8 min for a screening DBT study which represented a 47% increase over FFDM.^6^

Although we demonstrated changes in blink behaviour associated with fatigue, we did not observe a corresponding change in diagnostic accuracy over the 40-case reporting exercise. This may be explained by one of the study’s limitations where the large volumes of data generated from recording eye tracking necessitated short reading breaks every 40 min to download data, which may have attenuated/reduced the effects of fatigue in readers. However, previous studies have demonstrated that fatigue in visual scenarios develops under 40 min. For instance, Zhang *et al* (2013) found that 3D viewing of movies, compared with 2D viewing, was associated with an increased level of alertness due to an increase in blink frequency amongst other factors. However, within 40 min of viewing, alertness decreased to suggest visual fatigue.^30^ Scheduling breaks into radiology reporting sessions has been suggested as an intervention to reduce fatigue, however, further focused work is needed to correlate fatigue characteristics with performance metrics in order to advise optimal reporting session duration, break frequency and break duration.

Another limitation of this study was the occurrence of missing eye tracking data due to technical issues with the eye tracker. This rendered the data from three participants uninterpretable, and thus their data were omitted from analysis. In addition, the findings of our study are only likely to be generalisable across readers in screening programmes where double reading is the standard of care.

In conclusion, significant changes in blinking characteristics over time were observed when reading a test series of 40 DBT cases. There was a significant increase in overall blink durations with session progression due to a significant decrease in the rate of ultra-short blinks of ≤50 ms and an increase in longer blinks of 51–100 ms duration. Though more work is required, these changes in blinking behaviours could potentially serve as objective markers for the onset of fatigue in medical image reporting sessions, which may prove useful for further research into developing reading protocols to maximise performance.

## Key points

- As 23 readers reported on a set of 40 DBT screening cases, readers’ average blink duration significantly increased as the reading session progressed (0.38 ms per case; *p* < 0.0001).
- A significant decrease in the frequency of ultra-short blinks (≤50 ms, *p* = 0.008) and a significant increase in the frequency of short blinks (between 51 and 100 ms, *p* = 0.0005) was identified.
- In line with other studies, these changes in blink characteristics seem indicative of fatigue onset in DBT screeners, but no associated change in diagnostic accuracy was observed.
